# Supplementary material for: Do Current Smokers and Ex-Smokers Who Use Nicotine Vaping Products Daily Versus Weekly Differ on Their Reasons for Vaping? Findings from the 2020 ITC Four Country Smoking and Vaping Survey
Source: Int J Environ Res Public Health. 2022 Oct 29;19(21):14130. doi: 10.3390/ijerph192114130 (PMC9653847; doi:10.3390/ijerph192114130)
Supplement: Supplementary file 1 [file ijerph-19-14130-s001.zip › ijerph-1966591-supplementary.pdf]

Figure S1. Study flow diagram (4CV3, 2020)

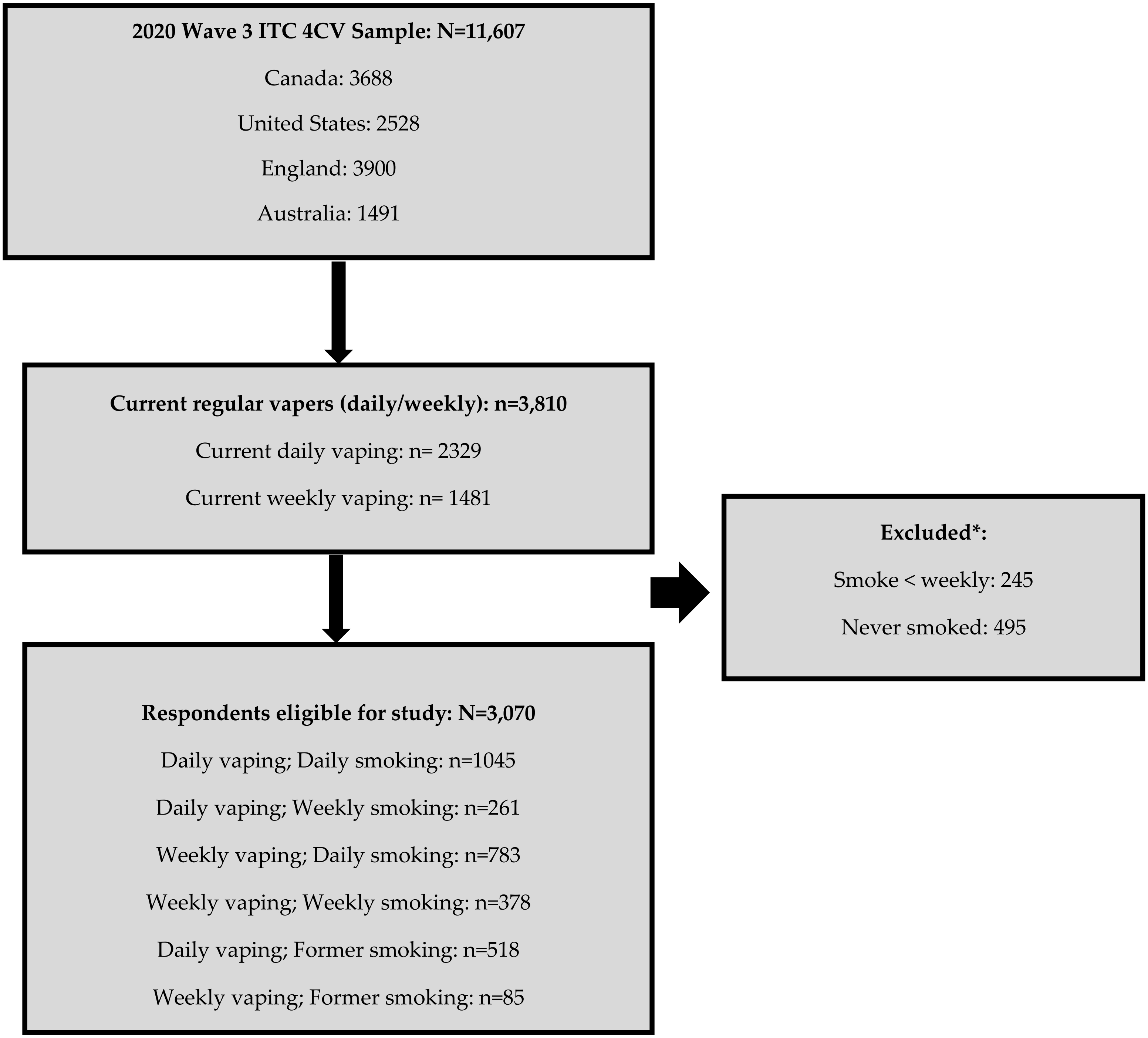

Supplemental Table S1. Reasons for vaping among daily/weekly vapers (current smokers and ex-smokers): ITC 4CV 2016 and 2020 Surveys

	2016* Weighted %		2020 Weighted %	
	**Current smokers**	**Ex-smokers**	**Current smokers**	**Ex-smokers**
Vaping is less harmful to me than smoking	Not assessed		70.9	92.7
Vaping is less harmful than smoking to other people around me	77.9	90.0	74.5	85.0
I enjoy vaping	69.6	90.6	78.2	93.7
I save money by vaping instead of smoking	68.3	89.5	67.0	89.7
I like the e-liquid flavours	Not assessed		71.9	78.9
Vaping is more acceptable than smoking	74.3	78.6	69.9	65.6
I can vape in places where I can't smoke	66.5	66.3	65.5	60.3
Vaping helps me cut down on the number of cigarettes I smoke	85.6	—	81.3	—
Vaping might help me stop smoking cigarettes	77.4	—	73.0	—
Vaping might help me stay quit from smoking cigarettes	—	88.3	—	92.3

*Published: Yong HH, Borland R, Cummings KM, Gravely S, Thrasher JF, McNeill A, Hitchman S, Greenhalgh E, Thompson ME, Fong GT. Reasons for regular vaping and for its discontinuation among smokers and recent ex-smokers: findings from the 2016 ITC Four Country Smoking and Vaping Survey. Addiction. 2019 Oct;114 Suppl 1(Suppl 1):35-48.

Supplemental Table S2. Logistic regression models on reasons for vaping among current smokers

n=2467		% yes (weighted)	95% CI	P-value (main effect)
**Vaping is less harmful to me than smoking**				
Vaping frequency	Daily	73.9	70.2-77.3	0.01
	Weekly	67.3	63.5-70.9	
Age	18-39	69.4	65.9-72.6	0.21
	40+	72.7	68.6-76.4	
Sex	Female	70.3	66.2-74.1	0.70
	Male	71.3	67.8-74.6	
Income	Low	65.8	60.1-71.1	0.01
	Moderate/high	72.9	69.9-75.7	
	Not reported	53.6	36.2-70.2	
Education	Low	72.7	66.0-78.5	0.27
	Moderate/high	70.9	68.0-73.6	
	Not reported	51.7	27.3-75.2	
Country	Australia	78.7	68.7-86.2	0.03
	Canada	70.3	66.5-73.9	
	England	73.0	68.7-76.9	
	US	63.4	56.7-69.6	
Smoking frequency	Daily	69.3	66.3-72.1	0.04
	Weekly	75.9	70.3-80.7	
**Vaping is less harmful than smoking to other people around me**				
Vaping frequency	Daily	77.4	74.0-80.6	0.01
	Weekly	70.8	67.0-74.4	
Age	18-39	69.8	66.3-73.1	<0.01
	40+	79.4	75.7-82.7	
Sex	Female	78.2	74.4-81.5	0.01
	Male	71.6	68.1-74.8	
Income	Low	73.7	68.3-78.5	0.07
	Moderate/high	75.2	72.2-78.0	
	Not reported	56.4	38.7-72.5	
Education	Low	72.3	64.9-78.6	0.50
	Moderate/high	75.0	72.2-77.6	
	Not reported	62.9	34.6-84.5	
Country	Australia	83.9	74.6-90.2	0.12
	Canada	75.4	71.8-78.6	
	England	73.6	69.3-77.5	
	US	70.9	64.3-76.8	
Smoking frequency	Daily	72.7	69.7-75.5	0.02
	Weekly	79.8	74.7-84.1	
**I enjoy vaping**				
Vaping frequency	Daily	83.6	80.5-86.4	<0.001
	Weekly	70.8	67.0-74.4	
Age	18-39	78.5	75.2-81.4	0.85
	40+	78.0	74.1-81.4	
Sex	Female	77.7	73.6-81.2	0.69
	Male	78.7	75.4-81.6	

n=2467		% yes (weighted)	95% CI	P-value (main effect)
Income	Low	76.8	71.3-81.5	0.53
	Moderate/high	78.4	75.5-81.1	
	Not reported	86.1	66.8-95.0	
Education	Low	80.7	74.0-86.0	0.32
	Moderate/high	78.0	75.3-80.5	
	Not reported	62.9	35.5-83.9	
Country	Australia	78.7	67.7-86.7	0.01
	Canada	83.6	80.5-86.3	
	England	75.8	71.5-79.6	
	US	76.2	69.8-81.6	
Smoking frequency	Daily	76.8	73.9-79.4	0.04
	Weekly	82.6	77.6-86.7	
**I save money by vaping instead of smoking**				
Vaping frequency	Daily	74.1	70.6-77.2	<0.001
	Weekly	58.3	54.2-62.3	
Age	18-39	62.0	58.4-65.5	<0.001
	40+	72.5	68.5-76.2	
Sex	Female	71.5	67.5-75.1	<0.01
	Male	63.6	59.9-67.1	
Income	Low	65.7	59.9-71.2	0.34
	Moderate/high	67.8	64.6-70.8	
	Not reported	54.6	36.0-72.0	
Education	Low	74.2	67.4-79.9	0.04
	Moderate/high	66.0	63.0-68.9	
	Not reported	45.8	18.4-76.1	
Country	Australia	82.4	73.3-88.8	0.001
	Canada	66.4	62.4-70.2	
	England	67.9	63.6-72.0	
	US	58.1	51.2-64.8	
Smoking frequency	Daily	67.2	64.1-70.1	0.79
	Weekly	66.3	60.6-71.6	
**I like the e-liquid flavours**				
Vaping frequency	Daily	75.4	71.9-78.6	<0.01
	Weekly	67.5	63.6-71.3	
Age	18-39	74.8	71.4-77.9	0.01
	40+	68.1	63.9-72.0	
Sex	Female	71.9	67.7-75.8	0.98
	Male	71.8	68.4-75.0	
Income	Low	71.9	66.2-77.0	0.27
	Moderate/high	72.3	69.2-75.1	
	Not reported	56.7	36.5-74.9	
Education	Low	76.2	69.5-81.9	0.35
	Moderate/high	71.2	68.3-73.9	
	Not reported	64.9	35.6-86.1	
Country	Australia	76.1	66.7-83.5	0.05
	Canada	75.1	71.3-78.6	
	England	72.1	67.7-76.0	
	US	65.4	58.6-71.7	
Smoking frequency	Daily	70.0	66.9-72.9	0.02

n=2467		% yes (weighted)	95% CI	P-value (main effect)
	Weekly	77.6	72.3-82.1	
**Vaping is more acceptable than smoking**				
Vaping frequency	Daily	72.2	68.6-75.6	0.06
	Weekly	67.3	63.4-71.0	
Age	18-39	67.3	63.7-70.6	0.03
	40+	73.0	69.0-76.6	
Sex	Female	72.5	68.4-76.2	0.10
	Male	68.1	64.5-71.4	
Income	Low	70.8	65.3-75.9	0.83
	Moderate/high	69.8	66.7-72.7	
	Not reported	65.3	45.1-81.1	
Education	Low	66.3	58.8-73.1	0.20
	Moderate/high	70.8	67.9-73.5	
	Not reported	49.3	22.0-77.1	
Country	Australia	72.0	61.9-80.2	0.27
	Canada	66.8	62.8-70.5	
	England	69.6	65.2-73.6	
	US	73.5	66.9-79.1	
Smoking frequency	Daily	69.7	66.6-72.6	0.70
	Weekly	70.9	65.4-75.7	
**I can vape in places where I can't smoke**				
Vaping frequency	Daily	67.2	63.5-70.8	0.18
	Weekly	63.6	59.6-67.4	
Age	18-39	62.2	58.6-65.7	0.01
	40+	69.4	65.3-73.2	
Sex	Female	68.9	64.7-72.7	0.03
	Male	63.0	59.4-66.5	
Income	Low	65.4	59.6-70.7	0.90
	Moderate/high	65.7	62.4-68.8	
	Not reported	61.9	44.5-76.7	
Education	Low	59.1	51.5-66.4	0.07
	Moderate/high	66.8	63.9-69.6	
	Not reported	47.2	22.3-73.6	
Country	Australia	59.3	48.7-69.0	<0.001
	Canada	58.5	54.4-62.4	
	England	65.1	60.6-69.3	
	US	75.6	69.6-80.7	
Smoking frequency	Daily	65.9	62.8-68.9	0.60
	Weekly	64.2	58.5-69.6	
**Vaping helps me cut down on the number of cigarettes I smoke**				
Vaping frequency	Daily	85.1	82.2-87.6	<0.001
	Weekly	76.2	72.3-79.6	
Age	18-39	75.6	72.3-78.7	<0.001
	40+	86.7	83.4-89.4	
Sex	Female	84.9	81.5-87.8	<0.01
	Male	78.3	75.0-81.3	
Income	Low	81.8	76.8-85.9	0.96
	Moderate/high	81.1	78.3-83.6	
	Not reported	80.8	62.5-91.4	

n=2467		**% yes (weighted)**	**95% CI**	**P-value (main effect)**
Education	Low	85.8	79.8-90.2	0.12
	Moderate/high	80.7	78.0-83.0	
	Not reported	63.3	30.2-87.3	
Country	Australia	87.7	78.7-93.2	0.28
	Canada	82.4	79.2-85.2	
	England	80.4	76.4-83.8	
	US	78.9	72.6-84.0	
Smoking frequency	Daily	80.4	77.6-82.9	0.14
	Weekly	84.2	79.6-87.9	
**Vaping might help me quit smoking**				
Vaping frequency	Daily	76.7	73.2-79.8	<0.01
	Weekly	68.5	64.5-72.2	
Age	18-39	67.6	64.0-70.9	<0.001
	40+	78.7	75.0-82.1	
Sex	Female	76.2	72.3-79.7	0.03
	Male	70.5	67.0-73.8	
Income	Low	73.0	67.5-77.9	0.99
	Moderate/high	73.0	69.9-75.9	
	Not reported	72.0	54.1-84.9	
Education	Low	76.9	70.1-82.6	0.36
	Moderate/high	72.5	69.6-75.2	
	Not reported	62.1	31.9-85.1	
Country	Australia	79.8	70.2-86.9	0.11
	Canada	75.3	71.6-78.6	
	England	72.6	68.4-76.5	
	US	67.9	60.9-74.2	
Smoking frequency	Daily	71.6	68.6-74.5	0.05
	Weekly	77.2	72.2-81.6	

CI: Confidence interval. US: United States. P-value is the main effect of each variable in the model (analysis of effects).

Supplemental Table S3. Logistic regression models on reasons for vaping among ex-smokers

n=603		% yes (weighted)	95% CI	P-value (main effect)
**Vaping is less harmful to me than smoking**				
Vaping frequency	Daily	93.7	90.1-96.1	0.02
	Weekly	84.0	71.2-91.7	
Age	18-39	90.7	83.5-95.0	0.28
	40+	94.0	89.9-96.5	
Sex	Female	89.8	85.1-93.1	0.11
	Male	94.1	89.4-96.8	
Income	Low	92.6	88.0-95.5	0.98
	Moderate/high	92.8	87.9-95.8	
	Not reported	91.5	67.4-98.2	
Education	Low	93.7	86.5-97.2	0.86
	Moderate/high	92.2	87.9-95.1	
	Not reported	94.6	61.3-99.5	
Country	Australia	97.6	92.8-99.2	0.01
	Canada	92.0	83.8-96.3	
	England	94.2	88.5-97.2	
	US	85.0	76.5-90.7	
Quit duration	> 2 years	94.7	90.5-97.1	0.07
	≤ 2 years	90.3	85.0-93.9	
**Vaping is less harmful than smoking to other people around me**				
Vaping frequency	Daily	86.3	80.3-90.6	0.14
	Weekly	76.8	62.6-86.7	
Age	18-39	87.1	77.3-93.0	0.47
	40+	83.1	76.1-88.3	
Sex	Female	86.9	79.4-91.9	0.45
	Male	83.7	76.4-89.0	
Income	Low	75.6	64.3-84.2	0.05
	Moderate/high	86.9	80.5-91.4	
	Not reported	94.6	69.6-99.3	
Education	Low	84.6	72.5-92.0	0.98
	Moderate/high	85.2	79.4-89.6	
	Not reported	82.5	37.7-97.4	
Country	Australia	91.1	79.6-96.4	0.48
	Canada	88.0	77.5-94.0	
	England	82.1	72.2-89.0	
	US	84.8	76.3-90.6	
Quit duration	> 2 years	88.3	81.7-92.8	0.09
	≤ 2 years	81.6	74.3-87.3	
**I enjoy vaping**				
Vaping frequency	Daily	94.8	92.0-96.6	0.01
	Weekly	83.8	69.4-92.2	
Age	18-39	95.7	91.7-97.8	0.07
	40+	91.3	87.3-94.2	
Sex	Female	95.3	92.0-97.3	0.17
	Male	92.3	87.5-95.3	

n=603		% yes (weighted)	95% CI	P-value (main effect)
Income	Low	91.3	84.9-95.1	0.33
	Moderate/high	94.9	91.4-97.0	
	Not reported	89.3	62.5-97.7	
Education	Low	94.7	86.1-98.1	
	Moderate/high	88.3	83.4-91.9	
	Not reported	100.0	100.0-100.0	<0.001
Country	Australia	96.2	89.3-98.7	0.70
	Canada	94.3	89.0-97.1	
	England	92.5	87.3-95.7	
	US	94.3	88.6-97.3	
Quit duration	> 2 years	95.6	91.6-97.7	0.11
	≤ 2 years	91.5	86.9-94.6	
**I save money by vaping instead of smoking**				
Vaping frequency	Daily	90.5	86.3-93.6	0.18
	Weekly	84.5	72.5-91.9	
Age	18-39	86.0	77.6-91.7	0.09
	40+	92.2	87.9-95.1	
Sex	Female	88.2	80.7-93.1	0.45
	Male	90.7	86.2-93.8	
Income	Low	92.0	86.7-95.3	0.18
	Moderate/high	87.5	81.3-91.8	
	Not reported	96.0	77.4-99.4	
Education	Low	91.1	82.3-95.8	0.37
	Moderate/high	90.1	85.8-93.2	
	Not reported	63.1	15.4-94.1	
Country	Australia	97.5	84.5-99.7	0.03
	Canada	84.2	73.5-91.1	
	England	92.7	86.6-96.1	
	US	81.1	72.4-87.5	
Quit duration	> 2 years	91.9	86.4-95.3	0.19
	≤ 2 years	87.5	81.7-91.7	
**I like the e-liquid flavours**				
Vaping frequency	Daily	79.3	73.8--84.0	0.69
	Weekly	76.5	60.3-87.4	
Age	18-39	86.1	78.7-91.2	<0.01
	40+	70.9	63.5-77.3	
Sex	Female	79.4	73.1-84.5	0.86
	Male	78.6	71.0-84.6	
Income	Low	75.2	65.1-83.1	0.59
	Moderate/high	80.5	74.2-85.6	
	Not reported	78.1	55.7-91.0	
Education	Low	82.4	71.5-89.7	0.03
	Moderate/high	79.8	74.4-84.4	
	Not reported	26.1	5.3-68.8	
Country	Australia	80.6	65.8-90.0	0.49
	Canada	75.1	63.0-84.3	
	England	82.0	74.5-87.7	
	US	75.1	65.6-82.6	
Quit duration	> 2 years	79.3	70.5-86.0	0.90
	≤ 2 years	78.6	71.9-84.1	

n=603		% yes (weighted)	95% CI	P-value (main effect)
**Vaping is more acceptable than smoking**				
Vaping frequency	Daily	65.0	58.5-70.9	0.64
	Weekly	68.9	53.3-81.1	
Age	18-39	68.1	58.0-76.8	0.47
	40+	63.4	55.8-70.3	
Sex	Female	66.4	58.3-73.7	0.80
	Male	65.0	56.8-72.4	
Income	Low	61.5	50.2-71.7	0.17
	Moderate/high	65.0	57.6-71.7	
	Not reported	84.1	62.4-94.4	
Education	Low	66.6	54.8-76.6	0.97
	Moderate/high	65.5	58.9-71.5	
	Not reported	62.6	25.3-89.2	
Country	Australia	58.2	42.8-72.2	0.67
	Canada	62.5	51.4-72.4	
	England	67.3	56.7-76.3	
	US	67.5	58.0-75.8	
Quit duration	> 2 years	67.6	59.6-74.7	0.49
	≤ 2 years	63.8	55.6-71.3	
**I can vape in places where I can't smoke**				
Vaping frequency	Daily	60.5	54.1-66.5	0.89
	Weekly	59.3	43.5-73.3	
Age	18-39	61.6	51.3-70.9	0.73
	40+	59.2	51.2-66.7	
Sex	Female	59.5	51.7-66.8	0.81
	Male	60.8	52.4-68.6	
Income	Low	52.2	42.1-62.0	0.16
	Moderate/high	62.8	55.2-69.7	
	Not reported	69.9	44.7-87.0	
Education	Low	62.9	50.9-73.6	0.11
	Moderate/high	61.6	55.1-67.7	
	Not reported	21.3	5.0-58.3	
Country	Australia	37.8	24.5-53.3	0.02
	Canada	54.0	42.6-64.9	
	England	65.8	55.6-74.7	
	US	62.1	52.6-70.7	
Quit duration	> 2 years	55.8	46.6-64.6	0.19
	≤ 2 years	64.0	55.8-71.5	
**Vaping might help me stay quit from smoking cigarettes**				
Vaping frequency	Daily	93.0	89.3-95.4	0.29
	Weekly	87.9	71.4-95.5	
Age	18-39	89.0	81.2-93.8	0.16
	40+	94.4	89.2-97.2	
Sex	Female	95.4	90.7-97.8	0.04
	Male	89.3	83.4-93.3	
Income	Low	93.6	86.0-97.2	0.22
	Moderate/high	90.5	85.5-93.9	
	Not reported	98.0	87.3-99.7	

n=603		**% yes (weighted)**	**95% CI**	**P-value (main effect)**
Education	Low	93.2	82.9-97.5	0.05
	Moderate/high	92.8	88.7-95.5	
	Not reported	56.1	19.5-87.1	
Country	Australia	95.2	85.5-98.5	0.71
	Canada	89.6	79.8-95.0	
	England	92.7	85.4-96.5	
	US	92.7	86.1-96.3	
Quit duration	> 2 years	92.5	86.5-95.9	0.93
	≤ 2 years	92.2	87.2-95.3	

CI: Confidence interval. US: United States. P-value is the main effect of each variable in the model (analysis of effects). Results should be interpreted with caution due to small sample sizes (resulting in wide confidence intervals).
